# Первичный гиперальдостеронизм как самостоятельный фактор поражения почек. Потенциальные терапевтические стратегии и перспективы исследований

**DOI:** 10.14341/probl13679

**Published:** 2026-07-22

**Authors:** Н. М. Платонова, Р. М. Габибуллаев, Д. Р. Махачев, А. А.-Ю. Дотдуев, Д. А. Полякова, Д. А. Мухина, З. А. Бельбекова, В. В. Майер, А. С. Хадзиев, Е. А. Трошина

**Affiliations:** НМИЦ эндокринологии им. академика И.И. Дедова Россия; Endocrinology Research Centre Russian Federation; Российский национальный исследовательский медицинский университет им. Н.И. Пирогова (Пироговский Университет) Россия; Pirogov Russian National Research Medical University (Pirogov University), Ministry of Health of the Russian Federation Russian Federation; Ингушский государственный университет Россия; Ingush State University Russian Federation

**Keywords:** гиперальдостеронизм, почка, альдостерон, альбуминурия, скорость клубочковой фильтрации, антагонисты минералокортикоидных рецепторов, адреналэктомия, hyperaldosteronism, kidney, aldosterone, albuminuria, glomerular filtration rate, mineralocorticoid receptor antagonists, adrenalectomy

## Abstract

Первичный гиперальдостеронизм (ПГА) — наиболее частая из потенциально излечимых причин вторичной артериальной гипертензии и независимый фактор риска хронической болезни почек (ХБП). Обзор фокусируется на патофизиологических механизмах почечного поражения при ПГА, включая активацию минералокортикоидных рецепторов, негеномные сигнальные пути через GPER, усиление окислительного стресса и экспрессию провоспалительных и профибротических медиаторов (TGF-β1, NF-κB, NLRP3). Показано, что хронический избыток альдостерона инициирует повреждение подоцитов, микроваскулярную дисфункцию и интерстициальный фиброз, приводящие к снижению скорости клубочковой фильтрации и прогрессированию ХБП.

Обсуждаются генетические детерминанты (мутации KCNJ5, ATP1A1, CACNA1D), ассоциированные с более выраженным почечным ремоделированием, и диагностические биомаркеры (L-FABP, KIM-1, микроальбуминурия), отражающие ранние стадии повреждения нефронов. Сравнительный анализ хирургического и медикаментозного лечения демонстрирует, что адреналэктомия обеспечивает более стойкую нефропротекцию, тогда как терапия антагонистами минералокортикоидных рецепторов требует динамического контроля функции почек. Особое внимание уделено новым стратегиям таргетной терапии, включая нестероидные антагонисты и ингибиторы альдостеронсинтазы (баксдростат). Обзор объединяет молекулярные и клинические аспекты альдостерон-индуцированного почечного повреждения, подчеркивая значение ранней диагностики и персонализированных подходов к предотвращению прогрессирования ХБП у пациентов с ПГА.

## Abstract (en)

Primary aldosteronism (PA) is the most common curable cause of secondary hypertension and an independent risk factor for chronic kidney disease (CKD). This review focuses on the pathophysiological mechanisms of aldosterone-induced renal injury, including mineralocorticoid receptor activation, nongenomic signaling via GPER, oxidative stress, and the upregulation of profibrotic and inflammatory mediators such as TGF-β1, NF-κB, and NLRP3. Chronic aldosterone excess leads to podocyte injury, microvascular dysfunction, and interstitial fibrosis, resulting in decreased glomerular filtration rate and CKD progression.

Genetic mutations (KCNJ5, ATP1A1, CACNA1D) and tubular stress biomarkers (L-FABP, KIM-1, microalbuminuria) are discussed as key factors of early nephron injury. Comparative clinical data show that adrenalectomy provides superior nephroprotection compared to pharmacological blockade, while long-term therapy with mineralocorticoid receptor antagonists requires careful renal monitoring. Emerging therapeutic strategies, including nonsteroidal MR antagonists and aldosterone synthase inhibitors (baxdrostat), offer new prospects for targeted nephroprotection.

By integrating molecular and clinical evidence, this review highlights the central role of aldosterone in renal pathology and emphasizes early detection and personalized treatment approaches to prevent CKD progression in patients with PA.

## Введение

Первичный гиперальдостеронизм (ПГА) — наиболее частая из потенциально излечимых причин вторичной артериальной гипертензии и встречается, по разным данным, у 5–13% пациентов с повышенным артериальным давлением. Избыточная секреция альдостерона не только поддерживает неконтролируемую гипертензию, но и оказывает прямое негативное воздействие на органы-мишени, в первую очередь на почки. ПГА остается недодиагностированным у значительной части пациентов, особенно при сочетании с хронической болезнью почек (ХБП), что откладывает начало терапии и повышает риск прогрессирования нефропатии. Усиление внимания к этой проблеме обусловлено тем, что своевременная диагностика и лечение ПГА способны не только нормализовать артериальное давление, но и предотвратить развитие необратимых структурных изменений в почках, тем самым улучшая долгосрочный прогноз [1][2].

Цель данного обзора — представить современные данные о почечном поражении при ПГА, охватив ключевые патофизиологические механизмы, клинические проявления, результаты исследований, терапевтические подходы — от хирургического лечения до применения антагонистов минералокортикоидных рецепторов и новых таргетных стратегий — и определить перспективные направления дальнейших исследований.

## Патофизиология почечного повреждения при гиперальдостеронизме

Хронический избыток альдостерона при первичном гиперальдостеронизме вызывает выраженные функциональные и морфологические изменения в клубочковом аппарате. Активация минералокортикоидных рецепторов (МР) в нефроне и сосудистом эндотелии приводит к натрий- и объёмзависимой гиперволемии, снижению чувствительности тубулогломерулярной обратной связи и, как следствие, к гломерулярной гиперфильтрации — одному из ранних проявлений заболевания. На этом этапе часто выявляются повышенная скорость клубочковой фильтрации (СКФ) и микроальбуминурия, маскирующие начальное структурное повреждение.

Дополнительным фактором, играющим ключевую роль в патогенезе гиперфильтрации и задержки натрия, является влияние альдостерона на эпителиальные каналы и насосы, обеспечивающие транспорт электролитов в дистальных отделах нефрона. Через активацию минералокортикоидных рецепторов в главных клетках собирательных трубочек альдостерон усиливает экспрессию и активность эпителиального натриевого канала (ENaC), расположенного на апикальной мембране, что способствует реабсорбции натрия из просвета канальца. Образующийся трансэпителиальный градиент натрия поддерживает гиперволемию и внутриклубочковую гипертензию. На базолатеральной мембране в этом процессе участвует натрий-калиевый насос (Na⁺/K⁺-АТФаза), который активно транспортирует натрий в интерстициальное пространство в обмен на калий. При гиперальдостеронизме наблюдается повышение активности и плотности Na⁺/K⁺-АТФазы, что дополнительно усиливает задержку натрия и потерю калия. Эти процессы ведут к формированию гипокалиемии и нарушению тубулогломерулярной обратной связи, усиливая давление-зависимое повреждение почек [3].

Морфологические исследования почечных биоптатов у пациентов с ПГА демонстрируют, что даже при сохранной СКФ наблюдаются гипертрофия клубочков, утолщение капиллярных стенок, гиалиноз артериол и сегментарный гломерулосклероз. Ключевым звеном этих изменений считается повреждение подоцитов — клеток, обеспечивающих целостность фильтрационного барьера. Альдостерон нарушает организацию подоцитарного цитоскелета, повышает проницаемость базальной мембраны и способствует развитию альбуминурии. Нарушение эндотелиальной функции, в частности снижение активности эндотелиальной NO-синтазы (eNOS), усиливает локальную вазоконстрикцию эфферентных артериол и повышает внутриклубочковое давление, что дополнительно поддерживает гиперфильтрацию и ускоряет ремоделирование сосудистого русла. Морфологически этот процесс проявляется гипертрофией и склерозом клубочков, являющихся предикторами снижения фильтрационной функции и прогрессирования ХБП при ПГА [4][5] (рис. 1).

**Figure:**
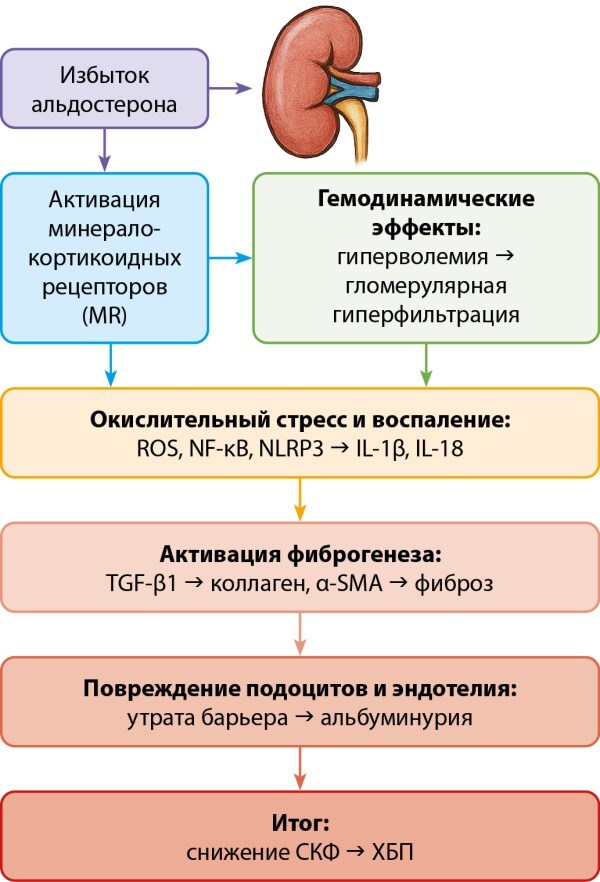
Рисунок 1. Патогенез почечного повреждения при первичном гиперальдостеронизме. Примечания: MR — минералокортикоидные рецепторы; ROS — активные формы кислорода; NF-κB — ядерный фактор каппа B; NLRP3 — NOD-подобный рецептор семейства NLR; IL-1β — интерлейкин-1β; IL-18 — интерлейкин-18; TGF-β1 — трансформирующий фактор роста β1; α-SMA — α-гладкомышечный актин; СКФ — скорость клубочковой фильтрации; ХБП — хроническая болезнь почек.

Независимо от уровня артериального давления, альдостерон оказывает прямое провоспалительное и профибротическое действие на почечную ткань. Активация МР в эпителии дистальных извитых канальцев стимулирует экспрессию генов провоспалительных цитокинов и компонентов внеклеточного матрикса, что приводит к ремоделированию интерстиция. Центральную роль в этом процессе играет трансформирующий фактор роста β1 (TGF-β1), активация которого при избытке альдостерона происходит как геномными, так и негеномными путями. Повышение экспрессии TGF-β1 инициирует путь Smad2/3, индуцирует фибробластную трансформацию и продукцию коллагена I и III типов, формируя интерстициальный фиброз [6].

Альдостерон стимулирует образование реактивных форм кислорода (ROS), активирует NF-κB и повышает секрецию хемокинов, включая MCP-1, что способствует инфильтрации макрофагов М1-фенотипа и поддерживает хроническое воспаление. В этих условиях формируется порочный круг: ROS-зависимая активация инфламмасомы NLRP3 усиливает секрецию IL-1β и IL-18, что способствует апоптозу канальцевых клеток и прогрессированию фиброза [7].

Наряду с классическими геномными эффектами альдостерон реализует негеномные действия, которые играют важную роль в патогенезе почечного и сосудистого повреждения. В отличие от медленно развивающихся геномных эффектов, опосредованных активацией ядерных минералокортикоидных рецепторов и транскрипцией генов, негеномные эффекты возникают в течение секунд–минут и не требуют синтеза новых белков. Эти быстрые сигнальные события активируются через мембранно-связанные рецепторы, включая G-белок-связанные рецепторы, такие как GPER (G-protein-coupled estrogen receptor), но также и иные рецепторы — например, рецепторы к эпидермальному фактору роста (EGFR), рецепторы тирозинкиназного типа, и модифицированные формы МР, локализованные вне ядра [8].

Негеномные эффекты альдостерона включают быстрый приток ионов натрия и кальция, активацию вторичных мессенджеров (cAMP, IP3), стимуляцию сигнальных каскадов (PI3K/Akt, ERK1/2), а также модуляцию эндотелиальной функции. В частности, они приводят к активации NADPH-оксидазы, повышению продукции реактивных форм кислорода (ROS), снижению активности эндотелиальной NO-синтазы и вазоконстрикции. Эти процессы развиваются независимо от транскрипционных механизмов и могут усиливаться на фоне хронической стимуляции альдостероном. При этом их влияние на почечную гемодинамику и ремоделирование канальцев оказывается значимым даже при нормальном уровне артериального давления [9].

Важно, что негеномные эффекты альдостерона, в отличие от геномных, могут сохраняться при применении классических антагонистов минералокортикоидных рецепторов, таких как спиронолактон. Это связано с тем, что GPER и другие мембранные мишени не блокируются этими препаратами, что представляет собой потенциальную терапевтическую проблему при ПГА. Отсутствие ингибирования этих путей может способствовать продолжающемуся фиброзу, воспалению и нарушению ионного транспорта, несмотря на прием МР-блокаторов. Учитывая это, перспективным направлением является разработка препаратов, способных блокировать как геномные, так и негеномные эффекты альдостерона [10].

Наряду с избытком альдостерона, важную роль в патогенезе нефропатии при ПГА играет дисбаланс ренин-ангиотензин-альдостероновой системы (РААС), характеризующийся снижением активности ренина и уровня ангиотензина II. В нормальных условиях ангиотензин II регулирует тонус эфферентной артериолы и участвует в поддержании гломерулярной фильтрации, особенно при гиповолемии. При ПГА подавление ренина и ангиотензина II приводит к дисрегуляции афферентно-эфферентного баланса, снижению эфферентного сопротивления и развитию внутриклубочковой гипертензии.

На фоне низкого ангиотензина II уменьшается контррегуляция эффекта альдостерона, что способствует его преобладанию в тканях-мишенях. Это состояние, получившее название «Автономная гиперсекрекция альдостерона» (inappropriate aldosterone excess), усиливает минералокортикоидное действие даже при нормальном или погранично повышенном уровне гормона. Такой дисбаланс способствует нарушению тубулогломерулярной обратной связи, ухудшает ауторегуляцию почечного кровотока и ускоряет структурное повреждение нефронов. Помимо этого, снижение ангиотензина II сопровождается угнетением продукции вазоактивных простагландинов, дополнительно снижая компенсаторные механизмы, защищающие почки от гипоперфузии. В исследовании подчеркивается, что неадекватно высокий уровень альдостерона при низком ренине усиливает профибротические сигнальные каскады и способствует накоплению внеклеточного матрикса, что ускоряет прогрессирование интерстициального фиброза [11].

Эти патофизиологические изменения находят отражение в клинических наблюдениях, демонстрирующих связь между уровнем альдостерона и признаками раннего почечного повреждения у пациентов с ПГА. Клинические наблюдения Nakamura и соавт. (2021, Clinical Endocrinology) подтверждают, что повышение уровня альдостерона в плазме коррелирует с биохимическими маркерами тубулярного и гломерулярного повреждения у пациентов с первичным гиперальдостеронизмом, что указывает на клиническую значимость этих механизмов. Дополнительным фактором прогрессирования почечного поражения выступает гипокалиемия, усугубляющая эндотелиальную дисфункцию, снижающая почечный кровоток и вызывающая ишемические изменения паренхимы. Совокупность МР-зависимых и GPER-опосредованных сигналов, ROS-индуцированного стресса и хронического воспаления формирует патофизиологическую основу тубулоинтерстициального фиброза и утраты функции нефронов [12].

## Генетические и молекулярные факторы

Современные молекулярно-генетические данные свидетельствуют, что при первичном гиперальдостеронизме, связанном с определенными соматическими мутациями в клетках клубочковой зоны надпочечников — прежде всего KCNJ5, ATP1A1 и CACNA1D, — выраженность сосудистого и почечного повреждения может быть выше, чем при спорадических формах заболевания. В обзоре Turcu и соавт. (2022, Nature Reviews Endocrinology) подчеркивается, что эти мутации ассоциированы с гиперсекрецией альдостерона, активацией минералокортикоидных рецепторов и усиленным ремоделированием тканей-мишеней, что создает предпосылки для раннего структурного повреждения почек [13].

На этом фоне особое внимание привлекают исследования, направленные на выявление молекулярных маркеров скрытого почечного поражения. У пациентов с ПГА отмечаются повышенные уровни мочевых маркеров канальцевого повреждения — N-ацетил-β-D-глюкозаминидазы, L-FABP и KIM-1, — а также микроальбуминурия при сохранной скорости клубочковой фильтрации. Эти изменения рассматриваются как проявление субклинического поражения канальцевого эпителия, часть которых обратима после устранения гиперальдостеронизма.

В проспективной работе Wu и соавт. (2023, Journal of the American Heart Association) установлено, что исходная концентрация L-FABP в моче обладает прогностическим значением: более высокие ее уровни ассоциировались с риском снижения скорости клубочковой фильтрации в течение 12 месяцев после лечения. Эти данные подчеркивают роль L-FABP как потенциального маркера ранней диагностики и стратификации риска прогрессирования хронической болезни почек у пациентов с ПГА [14].

## Клинические проявления почечного повреждения при ПГА

Поражение почек при первичном гиперальдостеронизме часто проявляется микроальбуминурией, даже при нормальной или повышенной СКФ. У пациентов с ПГА экскреция альбумина с мочой достоверно выше, чем при эссенциальной гипертензии аналогичной степени, что отражает нарушение целостности клубочкового фильтра под действием избытка альдостерона и внутриклубочковой гиперфильтрации. После устранения гиперальдостеронизма — хирургическим путем или назначением антагонистов минералокортикоидных рецепторов (АМР) — альбуминурия обычно существенно снижается, что подтверждает обратимость ранних изменений [15].

У многих пациентов после лечения наблюдается временное снижение СКФ — так называемый дип фильтрации. Это не свидетельствует о почечном повреждении, а отражает нормализацию ранее существовавшей гиперфильтрации. Чем выраженнее этот спад после коррекции гиперальдостеронизма, тем благоприятнее последующий прогноз: скорость снижения функции почек в динамике замедляется. Таким образом, умеренное (на 10–15%) снижение СКФ в сочетании с уменьшением альбуминурии расценивается как признак восстановления гломерулярной авторегуляции и нефропротективного эффекта терапии [16].

Как показано в исследовании Fernández-Argüeso и соавт. (2021, Endocrine), пациенты с первичным гиперальдостеронизмом имеют значительно более высокий риск развития и прогрессирования хронической болезни почек по сравнению с больными эссенциальной гипертензией. У них чаще выявляются сниженная СКФ (<60 мл/мин/1,73 м²), повышение уровня креатинина и более быстрый темп ее снижения. Эти данные подтверждают, что ПГА является независимым фактором риска прогрессирования ХБП, обусловленным не только повышенным артериальным давлением, но и прямыми нефротоксическими эффектами альдостерона [17].

Помимо гемодинамических нарушений, ключевую роль играет сосудистая дисфункция. Альдостерон снижает экспрессию эндотелиальной NO-синтазы (eNOS) и нарушает регуляцию сосудистого эндотелиального фактора роста (VEGF). На ранних стадиях избыток гормона вызывает гиперэкспрессию VEGF и повышение проницаемости капилляров, тогда как при стойком оксидативном стрессе наблюдается подавление ангиогенеза и апоптоз эндотелиоцитов. Эти процессы приводят к нарушению микроангиогенеза, интерстициальной гипоксии и ремоделированию сосудов, формируя морфологическую основу хронического фиброза почечной паренхимы [18].

Даже после успешного устранения гиперальдостеронизма у части пациентов функция почек продолжает снижаться. Метаанализ показал, что долгосрочная динамика СКФ после лечения зависит от возраста, пола, исходного уровня давления, концентрации калия и степени контроля артериальной гипертензии. Сохранение повышенного давления после терапии является ключевым прогностическим фактором ухудшения функции почек. В исследовании Haze и соавт. (2022, Journal of Human Hypertension) достижение целевого систолического давления <130 мм рт.ст. через шесть месяцев после лечения ПГА ассоциировалось с более благоприятной динамикой СКФ [19].

Кроме того, у пациентов с ПГА часто наблюдается нарушение циркадного профиля давления («non-dipper»-паттерн) с отсутствием ночного снижения и повышенной ночной нагрузкой, что дополнительно ускоряет почечное повреждение. Суточное мониторирование демонстрирует более высокие среднесуточные и ночные значения давления у больных ПГА по сравнению с эссенциальной гипертензией при сопоставимой терапии. Эти данные подчёркивают необходимость строгого контроля артериального давления, регулярного мониторинга альбуминурии и оценки СКФ для раннего выявления прогрессирования ХБП у пациентов с ПГА [20].

Дополнительными проявлениями ПГА, связанными с нарушением водно-электролитного баланса, могут быть полиурия и никтурия. Эти симптомы обусловлены сочетанием факторов: повышенной фильтрационной нагрузкой, гипокалиемией, нарушающей концентрационную способность почек, и снижением секреции антидиуретического гормона в условиях гиперволемии. Нарушение функции собирательных трубочек, снижение чувствительности к вазопрессину и гипостенурия способствуют учащенному ночному диурезу, что может снижать качество жизни пациентов и требует внимания при клиническом обследовании [21][22].

## Лечение: хирургическое и медикаментозное — влияние на почки и побочные эффекты

Основными стратегиями лечения первичного гиперальдостеронизма являются хирургическое удаление источника гиперсекреции альдостерона (адреналэктомия при односторонней аденоме) и длительная медикаментозная терапия антагонистами минералокортикоидных рецепторов при двусторонней гиперплазии или невозможности оперативного вмешательства. Оба подхода эффективно снижают артериальное давление и уменьшают поражение органов-мишеней, однако их влияние на почки различается.

Хирургическое лечение устраняет причину гиперальдостеронизма и нормализует уровень гормона, что сопровождается кратковременным снижением СКФ вследствие устранения гиперфильтрации. Медикаментозная терапия блокирует эффекты альдостерона, но не подавляет его продукцию, что ведет к сохранению резидуальной гормональной активности.

Сравнительные наблюдения показали, что в долгосрочной перспективе темпы снижения СКФ после адреналэктомии ниже, чем при терапии АМР. Среднегодовое падение СКФ после хирургического лечения составляет около −0,8 мл/мин/1,73 м², что сопоставимо с показателями при эссенциальной гипертензии, тогда как при длительном приёме спиронолактона — около −1,6 мл/мин/1,73 м². За десятилетний период частота терминальной почечной недостаточности в группе медикаментозной терапии почти в два раза выше [23].

Совокупность данных свидетельствует, что полное устранение избытка альдостерона хирургическим путем обеспечивает более выраженную нефропротекцию, чем его фармакологическая блокада. Однако прямых рандомизированных сравнений пока нет, и выбор метода лечения определяется латеральностью процесса и общим состоянием пациента. На основании данных проспективных и обзорных исследований, проведенных Ahmed и соавт. (2022), представлена сравнительная характеристика влияния хирургического и медикаментозного лечения с использованием АМР на функцию почек у пациентов с первичным гиперальдостеронизмом на примере спиронолактона (таблица 1).

**Table:** Таблица 1. Влияние стратегий лечения на функцию почек при ПГА

Показатель	Адреналэктомия	Спиронолактон
СКФ (динамика)	Резкое снижение СКФ в первые месяцы (≈8 мл/мин/1,73 м²), затем стабилизация	Постепенное уменьшение СКФ; годовое снижение –1,6 мл/мин/1,73 м², что превышает показатели при эссенциальной гипертензии
Альбуминурия	Значительное уменьшение альбуминурии (медиана 46 → 6 мг/г)	Умеренное снижение альбуминурии; статистически значимое улучшение не всегда достигается
Риск гиперкалиемии	Низкий; гипокалиемия исчезает после операции	Высокий; требует регулярного мониторинга калия; риск тяжелой гиперкалиемии повышается в 1,4 раза
Долгосрочные исходы	Меньший риск ХБП; улучшение контроля давления; возможна гиперурикемия и снижение СКФ >25% в 33% случаев	Риск прогрессирования ХБП выше; АМР обеспечивают нефропротекцию, но при недостаточном титровании возможно сохраняющееся повреждение

Как показано в международном когортном исследовании Monticone и соавт. (2025, Lancet Diabetes & Endocrinology), спиронолактон остается основным препаратом медикаментозной терапии первичного гиперальдостеронизма. Он эффективно снижает артериальное давление и устраняет гипокалиемию у большинства пациентов с двусторонней гиперплазией надпочечников. Поддерживающие дозы обычно составляют 25–50 мг/сут, однако повышение доз ограничивается риском побочных эффектов. Наиболее частыми нежелательными явлениями терапии спиронолактоном являются антиандрогенные эффекты — гинекомастия, снижение либидо у мужчин и менструальные нарушения у женщин. В упомянутом исследовании гинекомастия наблюдалась примерно у 6–8% мужчин, особенно при дозах ≥50 мг/сут. Еще одним значимым осложнением является гиперкалиемия: при сохранной функции почек тяжёлые формы встречаются редко, однако легкое повышение концентрации калия регистрируется у 15–20% пациентов, что требует регулярного контроля электролитов и почечной функции [24].

К факторам риска гиперкалиемии относятся исходная хроническая болезнь почек, сахарный диабет, пожилой возраст и сопутствующий прием ингибиторов ренин-ангиотензиновой системы. Эти наблюдения согласуются с данными Bakris и соавт. (2020, New England Journal of Medicine), где в исследовании FIDELIO-DKD было показано, что аналогичные факторы повышают риск гиперкалиемии при терапии антагонистами минералокортикоидных рецепторов. В связи с этим контроль калия и СКФ рекомендуется проводить через 1–2 недели после начала лечения, затем на 8–12‑й неделе и далее каждые 3–6 месяцев, в зависимости от риска [25].

Как показывают наблюдения, преимущества адреналэктомии перед длительной медикаментозной терапией АМР особенно очевидны у пациентов с ПГА и сопутствующей хронической болезнью почек, где риск гиперкалиемии ограничивает возможность полноценной блокады МР [23]. У данной категории больных риск развития гиперкалиемии существенно ограничивает возможности достижения полноценной блокады МР с помощью фармакотерапии. Клинические данные и результаты обзорных исследований свидетельствуют о том, что длительная терапия спиронолактоном сопряжена с риском развития дозозависимых эндокринных нежелательных явлений (таких как гинекомастия, нарушения менструального цикла), а также гиперкалиемии. Риск гиперкалиемии особенно возрастает на фоне снижения СКФ, что нередко диктует необходимость коррекции терапии — снижения дозы или перехода на селективный блокатор рецепторов альдостерона, эплеренон, обладающий более благоприятным профилем переносимости [26][27].

Особое внимание в контексте безопасности терапии заслуживает внимание частота развития гиперкалиемии у пациентов с ПГА и сопутствующей ХБП. Метаанализ данных клинических наблюдений показал, что кумулятивная частота дозозависимой гиперкалиемии, явившейся основанием для пересмотра схемы лечения спиронолактоном, в исследуемой когорте пациентов с ПГА составляет 5–20% [24]. У пациентов с ХБП риск существенно выше: согласно консенсусу KDIGO, снижение СКФ <45 мл/мин увеличивает вероятность гиперкалиемии при приеме АМР в 2–3 раза, а уровень калия ≥5,5 ммоль/л регистрируется до 20–30% случаев в зависимости от дозировки и сопутствующей терапии [28]. Таким образом, сочетание ПГА и ХБП формирует когорту наибольшего риска развития гиперкалиемии на фоне терапии антагонистами минералокортикоидных рецепторов (АМР). Данная категория пациентов требует особенно тщательного мониторинга уровня калия и осторожного, постепенного титрования дозы препарата.

У пациентов с ХБП и сердечно-сосудистой коморбидностью ключевую роль в коррекции сохраняющейся гиперкалиемии приобретают современные калий-связывающие препараты — патиромер и циклосиликат циркония натрия. Их применение позволяет не отменять жизненно важную терапию ингибиторами ренин-ангиотензин-альдостероновой системы (РААС) и АМР, а оптимизировать и поддерживать ее на эффективном уровне [28][29].

В контексте подбора терапии АМР важным становится не только контроль электролитов, но и профиль переносимости конкретного препарата. Среди стероидных антагонистов минералокортикоидных рецепторов эплеренон рассматривается как альтернатива спиронолактону, обладающая более высокой селективностью к МР и сниженной активностью в отношении андрогенных и прогестагенных рецепторов. Это позволяет избежать таких побочных эффектов, как гинекомастия, снижение либидо или менструальные нарушения, характерные для терапии спиронолактоном. В недавнем исследовании Goi и соавт. (2025, The Journal of Clinical Endocrinology & Metabolism) было проведено сравнение эплеренона и спиронолактона у пациентов с первичным гиперальдостеронизмом до проведения адреналэктомии. Авторы показали, что долгосрочные почечные и клинические исходы в обеих группах были сопоставимы, однако эплеренон демонстрировал лучшую переносимость. Это прежде всего объяснялось более высокой селективностью эплеренона к минералокортикоидным рецепторам и отсутствием значимого взаимодействия с андрогеновыми и прогестагенными рецепторами, что снижало частоту гинекомастии, снижения либидо и менструальных нарушений по сравнению со спиронолактоном [26][27].

Тем не менее даже у препаратов с улучшенным профилем переносимости, таких как эплеренон, сохраняются ограничения — прежде всего риск гиперкалиемии, особенно у пациентов с сопутствующей ХБП. В связи с этим в последние годы активное внимание привлекают нестероидные антагонисты минералокортикоидных рецепторов нового поколения, прежде всего финеренон, обладающий иным механизмом связывания с рецептором и более благоприятными фармакокинетическими свойствами. По сравнению со стероидными АМР, финеренон демонстрирует более сбалансированное распределение между тканями, что снижает риск гиперкалиемии и гормональных побочных эффектов при сопоставимой эффективности.

В крупных рандомизированных исследованиях FIDELIO-DKD и FIGARO-DKD, проведенных у пациентов с диабетической ХБП, финеренон продемонстрировал достоверное снижение альбуминурии, замедление снижения СКФ, а также снижение риска сердечно-сосудистых событий. Эти данные легли в основу обновленных рекомендаций Американской диабетологической ассоциации (ADA), где финеренон рекомендован в качестве второй линии терапии у пациентов с сахарным диабетом 2 типа и ХБП на фоне базовой терапии ингибиторами РААС [30].

Несмотря на выраженные нефропротективные свойства финеренона, описанные преимущественно у пациентов с диабетической ХБП, данные о его применении непосредственно при первичном гиперальдостеронизме крайне ограниченны. На сегодняшний день опубликованы лишь отдельные клинические наблюдения. В одном из них финеренон назначался пациенту с ПГА при непереносимости спиронолактона и эплеренона и приводил к умеренному увеличению активности ренина и уровня калия, однако контроль артериального давления оставался недостаточным [31]. В другом наблюдении после адреналэктомии и повторной гиперальдостеронемии использование финеренона позволило стабилизировать уровень калия и улучшить показатели давления, что подчеркивает потенциальную роль препарата у пациентов с ограниченными альтернативами терапии [32]. Эти наблюдения подтверждают возможность применения финеренона в отдельных клинических ситуациях, однако доказательной базы для его рутинного использования при ПГА пока недостаточно. На основании данных международных когортных исследований и клинических наблюдений были сформулированы практические рекомендации по применению спиронолактона у пациентов с первичным гиперальдостеронизмом (табл. 2).

**Table:** Таблица 2. Практические рекомендации по терапии спиронолактоном

Параметр	Рекомендации
Стартовая доза	12,5 мг/сут у пациентов с нормальным уровнем калия и СКФ>45 мл/мин/1,73 м²; при тяжелой гипокалиемии начинать с 25 мг/сут
Титрация	Увеличивать дозу каждые 2–4 недели, ориентируясь на артериальное давление, уровень калия и активность ренина; целевой ориентир — выход ренина из супрессии (выше нижней границы нормы)
Мониторинг	Базовая оценка перед началом терапии → повтор через 1–2 недели у пациентов группы риска → универсальный контроль на 8–12‑й неделе → далее каждые 3 мес (при низком риске допустимо каждые 3–6 мес); обязательный контроль при каждой титрации дозы [28]
Пороговые значения K⁺/СКФ	При калии 5,5–6,0 ммоль/л — уменьшить дозу и исключить препараты, повышающие калий; при калии >6,0 ммоль/л — прекратить терапию. При СКФ<45 мл/мин/1,73 м² — использовать минимальные дозы и более частый контроль [25][28]
Действия при гиперкалиемии	Отменить или снизить дозу спиронолактона, использовать калийсвязывающие препараты, назначить диету с ограничением калия, возобновить терапию после нормализации показателей [28]

Однако выбор конкретного антагониста минералокортикоидных рецепторов должен основываться не только на эффективности, но и на профиле безопасности и переносимости. В клинической практике все чаще возникает необходимость сравнения спиронолактона с альтернативными препаратами — эплереноном и финереноном, которые различаются по степени селективности, частоте побочных эффектов и риску электролитных нарушений.

Ниже представлена сводная таблица, отражающая ключевые различия между основными АМР, применяемыми или обсуждаемыми при ПГА, и позволяющая провести их сравнительную оценку (табл. 3).

**Table:** Таблица 3. Сравнительная характеристика антагонистов минералокортикоидных рецепторов

Препарат	Спиронолактон	Эплеренон
Тип молекулы	Стероидный	Стероидный
Селективность к МР	Низкая (взаимодействует с андрогеновыми и прогестагенными рецепторами)	Более высокая, чем у спиронолактона
Гормональные побочные эффекты	Частые (гинекомастия, снижение либидо, менструальные нарушения)	Редкие
Риск гиперкалиемии	Выраженный, особенно при ХБП	Несколько ниже, чем у спиронолактона
Влияние на СКФ в динамике	Постепенное снижение, в среднем −1,6 мл/мин/1,73 м² в год	Сопоставимо со спиронолактоном
Эффект на альбуминурию	Умеренное снижение, не всегда статистически значимо	Сопоставимо
Доказанная кардионефропротекция	Нет	Нет
Основные ограничения	Гинекомастия, гиперкалиемия, ограниченная переносимость	Цена, необходимость 2-кратного приема

Несмотря на доказанную эффективность перечисленных выше препаратов, медикаментозная терапия не устраняет источник гиперсекреции альдостерона. Поэтому при односторонних формах заболевания хирургическое лечение остается методом выбора. Как отмечают Tang и соавт. (2021, Journal of the Endocrine Society), длительная терапия антагонистами минералокортикоидных рецепторов обеспечивает контроль артериального давления и коррекцию гипокалиемии, но у части пациентов сохраняется повышенная активность альдостерона и риск прогрессирования почечной дисфункции. В отличие от этого, адреналэктомия позволяет полностью устранить гормональную гиперпродукцию, что сопровождается стойкой нормализацией артериального давления, снижением альбуминурии и стабилизацией функции почек. В первые месяцы после операции возможно умеренное снижение СКФ, отражающее устранение гиперфильтрации, однако в дальнейшем темпы снижения функции почек становятся сопоставимыми с возрастной нормой [33].

Тем не менее у части пациентов сохраняется гипертензия, особенно при длительном анамнезе болезни или сопутствующем сахарном диабете. В этих случаях контроль давления остается ключевым детерминантом почечного прогноза. Поэтому после хирургического лечения необходим регулярный мониторинг функции почек, уровня калия и факторов метаболического риска, включая гиперурикемию, которая часто сопровождает ПГА и ухудшает почечные исходы [34][35].

## Перспективы и направления дальнейших исследований

На горизонте терапии первичного гиперальдостеронизма появились новые возможности, связанные с применением ингибиторов альдостеронсинтазы — препаратов, подавляющих синтез альдостерона в коре надпочечников. Как отмечает Ryu (2025, Endocrinology and Metabolism), этот класс средств способен изменить парадигму лечения, обеспечивая прямое воздействие на источник гиперсекреции гормона. В ранних клинических испытаниях Brown и соавт. (2025, New England Journal of Medicine) показали, что один из представителей группы, баксдростат, эффективно снижает уровень альдостерона и способствует нормализации артериального давления, позволяя уменьшить дозы сопутствующих антигипертензивных препаратов у большинства пациентов с ПГА. Подавление синтеза альдостерона потенциально может обеспечить нефропротекцию, сопоставимую с хирургическим устранением источника гиперсекреции. Однако остаются нерешенными вопросы безопасности, включая риск гиперкалиемии и возможной вторичной надпочечниковой недостаточности. В ближайшие годы требуется оценка влияния ингибиторов альдостеронсинтазы на почечные исходы и определение их места в терапевтической стратегии [36][37].

Ранняя диагностика почечного повреждения при ПГА остается сложной задачей, поскольку повышенная скорость клубочковой фильтрации вследствие гиперфильтрации может маскировать начальные структурные изменения. В этом контексте особое значение приобретают молекулярные и функциональные индикаторы, способные выявлять повреждение нефронов до развития выраженной протеинурии. Вариабельность артериального давления и отсутствие ночного снижения («non-dipper»-паттерн) у пациентов с ПГА тесно связаны с риском поражения органов-мишеней, включая почки, что подтверждает целесообразность интеграции параметров суточного профиля давления с биомаркерами канальцевого стресса и данными о генетических мутациях [38].

Создание многофакторных прогностических моделей позволит более точно оценивать индивидуальный риск прогрессирования хронической болезни почек и оптимизировать терапевтическую тактику. Стандартизированные протоколы наблюдения, включающие регулярную оценку СКФ, альбуминурии и воспалительных маркеров, должны стать неотъемлемой частью ведения пациентов с ПГА. Реализация таких подходов возможна лишь при тесном взаимодействии эндокринологов, нефрологов и кардиологов [39].

## Заключение

ПГА представляет собой не только одну из наиболее частых причин вторичной артериальной гипертензии, но и самостоятельный фактор почечного повреждения. Избыточная продукция альдостерона вызывает активацию минералокортикоидных рецепторов, развитие окислительного стресса, воспаления и фиброза, что приводит к гломерулярной гиперфильтрации, альбуминурии и постепенному снижению скорости клубочковой фильтрации.

После устранения гиперальдостеронизма — хирургическим путем или с применением АМР — наблюдается нормализация фильтрации и снижение альбуминурии. Хирургическое лечение обеспечивает наиболее полное восстановление гормонального баланса и ассоциируется с лучшими долгосрочными почечными исходами. Медикаментозная терапия спиронолактоном также демонстрирует выраженные нефропротективные эффекты, однако требует регулярного контроля калия и функции почек.

Появление новых нестероидных АМР, таких как финеренон, и ингибиторов альдостеронсинтазы, включая баксдростат, открывает возможности для повышения эффективности и безопасности лечения. Важным направлением остается ранняя диагностика и оценка индивидуального риска прогрессирования хронической болезни почек с использованием молекулярных маркеров (L-FABP, KIM-1) и генетических предикторов (например, мутаций KCNJ5).

Таким образом, своевременное выявление и коррекция гиперальдостеронизма позволяют предотвратить развитие необратимых изменений в почках и улучшить прогноз. Развитие персонализированных терапевтических стратегий и внедрение биомаркеров раннего поражения нефронов являются перспективными шагами к повышению эффективности нефропротекции у пациентов с ПГА.

## Дополнительная информация

Источники финансирования. Статья подготовлена при выполнении НИР в рамках государственного задания Минздрава России, рег. № 123021300098-7.

Конфликт интересов. Авторы декларируют отсутствие явных и потенциальных конфликтов интересов, связанных с содержанием настоящей статьи.

Участие авторов. Все авторы одобрили финальную версию статьи перед публикацией, выразили согласие нести ответственность за все аспекты работы, включая надлежащее изучение и решение вопросов, связанных с точностью или добросовестностью любой части исследования.
